# Early Outcomes of Minimally Invasive Right Anterior Thoracotomy *vs.* Median Full Sternotomy in Isolated Aortic Valve Replacement: A Propensity Score Analysis

**DOI:** 10.21470/1678-9741-2023-0108

**Published:** 2024-03-20

**Authors:** Anas O. Kh. Abubokha, Rui Li, Chen-he Li, Ahmad M. Zalloom, Xiang Wei

**Affiliations:** 1 Division of Cardiothoracic and Vascular Surgery, Tongji Hospital, Tongji Medical College, Huazhong University of Science and Technology, Wuhan, Hubei, People’s Republic of China; 2 Tongji Medical College, Huazhong University of Science and Technology, Wuhan, People’s Republic of China

**Keywords:** Aortic Valve, Cardiopulmonary Bypass, Thoracotomy, Mediastinitis, Operative Rime, Hospitalization, Drainage

## Abstract

**Introduction:**

This study aimed to compare the early postoperative outcomes of right anterior thoracotomy minimally invasive aortic valve replacement (RAT-MIAVR) surgery with those of median full sternotomy aortic valve replacement (MFS-AVR) approach with the goal of identifying potential benefits or drawbacks of each technique.

**Methods:**

This retrospective, observational, cohort study included 476 patients who underwent RAT-MIAVR or MFS-AVR in our hospital from January 2015 to January 2023. Of these, 107 patients (22.5%) underwent RAT-MIAVR, and 369 patients (77.5%) underwent MFS-AVR. Propensity score matching was used to minimize selection bias, resulting in 95 patients per group for analysis.

**Results:**

After propensity matching, two groups were comparable in preoperative characteristics. RAT-MIAVR group showed longer cardiopulmonary bypass time (130.24 ± 31.15 *vs.* 117.75 ± 36.29 minutes, *P*=0.012), aortic cross-clamping time (76.44 ± 18.00 *vs.* 68.49 ± 19.64 minutes, *P*=0.004), and longer operative time than MFS-AVR group (358.47 ± 67.11 minutes *vs.* 322.42 ± 63.84 minutes, *P*=0.000). RAT-MIAVR was associated with decreased hospitalization time after surgery, lower postoperative blood loss and drainage fluid, a reduced incidence of mediastinitis, increased left ventricular ejection fraction, and lower pacemaker use compared to MFS-AVR. However, there was no significant difference in the incidence of major complications and in-hospital mortality between the two groups.

**Conclusion:**

RAT-MIAVR is a feasible and safe alternative procedure to MFS-AVR, with comparable in-hospital mortality and early follow-up. This minimally invasive approach may be a suitable option for patients requiring isolated aortic valve replacement.

## INTRODUCTION

**Table:** 

Abbreviations, Acronyms & Symbols			
**ACC**	**= Aortic cross-clamping**	**FS**	**= Full sternotomy**
**AF**	**= Atrial fibrillation**	**HTK**	**= Histidine-tryptophan-ketoglutarate**
**AKI**	**= Acute kidney injury**	**ICU**	**= Intensive care unit**
**AV**	**= Aortic valve**	**LVEF**	**= Left ventricular ejection fraction**
**AVD**	**= Aortic valve disease**	**MFS**	**= Median full sternotomy**
**AVR**	**= Aortic valve replacement**	**MIAVR**	**= Minimally invasive aortic valve replacement**
**BMI**	**= Body mass index**	**MVR**	**= Mitral valve regurgitation**
**BP**	**= Bioabsorbable polymer**	**NYHA**	**= New York Heart Association**
**CAD**	**= Coronary artery disease**	**PSM**	**= Propensity score matching**
**CKD**	**= Chronic kidney disease**	**RAT**	**= Right anterior thoracotomy**
**COPD**	**= Chronic obstructive pulmonary disease**	**SD**	**= Standard deviation**
**CPB**	**= Cardiopulmonary bypass**	**SU**	**= Sutureless**
**EuroSCORE**	**= European System for Cardiac Operative Risk Evaluation**	**TIA**	**= Transient ischemic attack**

Ever since Dr. Harken and Starr introduced aortic valve replacement (AVR) surgery via median full sternotomy (MFS) in 1960^[1]^, the prognosis for aortic valve disease (AVD) has significantly improved, with a 60% to 80% increase in patients undergoing AVR surgery^[2]^. AVR via full sternotomy (FS) remains the golden standard, as indicated by a 2.6% in-hospital motility reported in the Society of Thoracic Surgeons database^[3]^. Throughout the years, surgeons worldwide have continuously pursued minimally invasive approaches to enhance the overall surgical method and improve postoperative quality of life with reduced tissue damage. The minimally invasive aortic valve replacement (MIAVR) was initially introduced in 1996, and it has emerged as a viable alternative to the FS approach. This innovation in surgical techniques offers the distinct advantage of reducing the degree of invasiveness inherent in the surgical procedure. It is noteworthy that this is accomplished without sacrificing the efficacy, quality, and safety particularly when performed in centers with extensive experience and advanced technique^[4-6]^. The right anterior thoracotomy (RAT) approach is a frequently employed minimally invasive approach that ensures the preservation of sternal stability. Potential advantages of RAT-MIAVR include cosmetic incisions, enhance safety for reoperation, and a decreased likelihood of sternal infection, all achieved without compromising the excellent results traditionally associated with MFS-AVR. However, some research studies have not been able to demonstrate the advantageous impact of RAT-MIAVR, with the exception of a reduced incision size^[7]^. Given the divergent conclusions from previous studies, the benefits of RAT-AVR remain unclear^[7,8]^. Our research aims to compare early postoperative outcomes of RAT-MIAVR with those of MFS-AVR to validate the efficacy of minimally invasive approach.

## METHODS

### Patients’ Selection and Data Collection

We retrospectively collected data of 476 patients diagnosed with isolated AVD who underwent AVR surgery within the period spanning from January 2015 to January 2023. Of these, 107 underwent RAT-MIAVR, while the remaining 369 were subjected to the conventional MFS-AVR. Data selection was guided by a variety of parameters including clinical relevance, patient demographics, preoperative risk factors, intraoperative parameters, postoperative outcomes, and data availability. This study was approved by the Ethics Committee of Tongji Hospital, affiliated with Tongji Medical College of Huazhong University of Science and Technology, which adheres to international ethical standards for conducting research involving human subjects and ensuring the integrity of the research (protocol number TJ-IRB202303103).

### Statistical Analysis

**Fig. 1:**
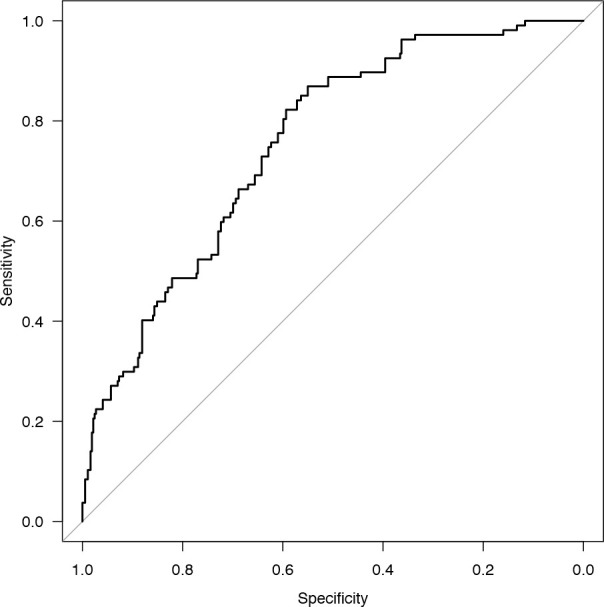
Receiver operating characteristic curve.

A comprehensive statistical evaluation was conducted to determine the association between preoperative parameters and the clinical outcomes of RAT-MIAVR and MFS-AVR procedures. In total, 26 preoperative variables were investigated, including age, sex, body mass index (BMI), blood pressure, bioabsorbable stent, chronic kidney disease, chronic obstructive pulmonary disease, diabetes, ejection fraction, endocarditis, European System for Cardiac Operative Risk Evaluation (or EuroSCORE) II, history of atrial fibrillation, height, history of alcohol consumption, history of coronary artery disease (CAD), history of transient ischemic attack, hypertension, New York Heart Association, obesity (BMI > 29), preoperative anemia, preoperative neurological complications, presence of functional mitral valve regurgitation, prior cardiac surgery, recent dialysis, smoking history, urgent operation, and weight. These variables served as the baseline for conducting both propensity score matching (PSM) and logistic regression analysis. The PSM was carried out through the Python-based software R Commander (version 1.78). An optimal caliper width^[9]^ of 0.2 was employed for the calculation and subsequent matching of propensity score variables. Pair matching was adjusted to achieve a 1:1 pair ratio, ultimately yielding a total sample population of 190. This population was evenly distributed between the two cohorts, with 95 participants (50%) assigned to the RAT-MIAVR group and 95 allocated to the MFS-AVR group (50%) (Table 1). In the process of conducting PSM, logistic regression analysis was performed, and a receiver operating characteristic curve was plotted (Figure 1). The area under the curve was determined to be 0.753 (95% confidence interval 0.705 - 0.802), indicating that the propensity score model exhibited a moderate-to-good discriminatory capacity between patients who underwent RAT-MIAVR and those subjected to MFSAVR.

**Table 1:** Preoperative characteristics.

Variables	Before PSM			After PSM		
	RAT-MIAVR (N=107)	MFS-AVR (N=369)	*P*-value	RAT-MIAVR (N=95)	MFS-AVR (N=95)	*P*-value
	Mean (± SD)/N (%)			Mean (± SD)/N (%)		
Age at surgery (years)	47.53 ± 14.23	50.49 ± 12.37	0.053	47.37 ± 14.01	48.09 ± 13.69	0.718
Female	25 (23.36)	109 (29.54)		24 (25.26)	23 (24.21)	
Male	82 (76.64)	260 (70.46)	0.211	71 (74.74)	72 (75.79)	0.866
Weight (kg)	67.33 ± 11.81	64.45 ± 10.95	0.019^*^	66.66 ± 11.46	67.16 ± 12.32	0.775
Height (m)	1.68 ± 0.09	1.68 ± 0.08	0.822	1.67 ± 0.08	1.68 ± 0.08	0.351
BMI (kg/m^2^)	24.00 ± 2.94	23.05 ± 3.08	0.005^**^	23.79 ± 2.96	23.60 ± 3.27	0.677
Obesity (BMI > 29)	2 (1.87)	8 (2.17)	0.849	2 (2.11)	1 (1.05)	0.561
Diabetes	8 (7.48)	43 (11.65)	0.219	8 (8.42)	6 (6.32)	0.579
Hypertension	30 (28.04)	122 (33.06)	0.326	27 (28.42)	29 (30.53)	0.750
Smoking history	31 (29.25)	95 (25.75)	0.472	25 (26.32)	29 (30.53)	0.520
Alcohol consumption	27 (25.23)	77 (20.87)	0.336	22 (23.16)	25 (26.32)	0.614
CKD	15 (14.02)	70 (18.97)	0.239	12 (12.63)	16 (16.84)	0.413
Recent dialysis	3 (2.80)	1 (0.27)	0.012^*^	1 (1.05)	0 (0.00)	0.316
Urgent operation	0 (0.00)	5 (1.36)	0.226	0 (0.00)	0 (0.00)	N/A
Previous cardiac surgery	8 (7.48)	28 (7.59)	0.969	6 (6.32)	10 (10.53)	0.296
History of TIA	1 (0.93)	7 (1.90)	0.495	1 (1.05)	2 (2.11)	0.561
History of CAD	10 (9.35)	70 (18.97)	0.019^*^	7 (7.37)	7 (7.37)	1.000
BP stented	3 (2.80)	10 (2.71)	0.958	2 (2.11)	3 (3.16)	0.650
Preoperative LVEF (%)	60.03 ± 8.52	59.83 ± 9.56	0.851	59.21 ± 8.32	60.53 ± 9.41	0.459
Presence of functional MVR	10 (9.35)	69 (18.70)	0.022^*^	10 (10.53)	8 (8.42)	0.620
NYHA I/II	89 (83.18)	310 (84.01)	0.837	32 (82.05)	66 (84.62)	0.723
NYHA II/IV	18 (16.82)	59 (15.99)	0.837	12 (15.38)	7 (17.95)	0.723
EuroSCORE II	0.02 ± 0.01	0.02 ± 0.01	0.227	0.02 ± 0.01	0.02 ± 0.01	0.970
COPD	17 (15.89)	51 (13.82)	0.591	17 (17.89)	18 (18.95)	0.852
Neurological disorders	8 (7.48)	8 (2.17)	0.007^**^	4 (4.21)	5 (5.26)	0.733
Preoperative anemia	6 (5.61)	37 (10.03)	0.160	5 (5.26)	2 (2.11)	0.248
History of AF	1 (0.93)	5 (1.36)	0.731	0 (0.00)	1 (1.05)	0.316
Endocarditis	4 (3.74)	54 (14.63)	0.002^**^	4 (4.21)	2 (2.11)	0.407

AF=atrial fibrillation; AVR=aortic valve replacement; BMI=body mass index; BP=bioabsorbable polymer; CAD=coronary artery disease; CKD=chronic kidney disease; COPD=chronic obstructive pulmonary disease; EuroSCORE=European System for Cardiac Operative Risk Evaluation; LVEF=left ventricular ejection fraction; MFS=median full sternotomy; MIAVR=minimally invasive aortic valve replacement; MVR=mitral valve regurgitation; NYHA=New York Heart Association; PSM=propensity score matching; RAT=right anterior thoracotomy; SD=standard deviation; TIA=transient ischemic attack

* *P*<0.05,

** *P*<0.01

The Chi-square (χ^2^) test and Student’s *t*-test (independent *t*-test) were employed to assess the significance of the relationships between preoperative variables and the outcomes of RAT-MIAVR and MFS-AVR for both binary and continuous data types. These analyses were carried out using the online Statistical Products and Service Solutions Automatically (or SPSSAU) software, version 22.0. Preoperative characteristics, as well as intraoperative and postoperative characteristics, were systematically arranged in distinct tables (Tables 1 to 3).

**Table 3:** Matched postoperative characteristics.

Variables	RAT-MIAVR (N=95)	MFS-AVR (N=95)	*P*-value
	Mean (± SD)/N (%)		
In-hospital stay after surgery (days)	14.78 ± 8.10	17.97 ± 7.49	0.005^**^
Length of ICU stay (days)	4.51 ± 5.23	3.77 ± 2.55	0.303
RBC, total, 1^st^ day (10^12^/L)^*^	3.51 ± 0.44	3.59 ± 0.56	0.249
Platelets, total, 1^st^ day (10^9^/L)^*^	125.68 ± 35.22	116.59 ± 44.83	0.122
Estimated blood loss during operation (mL)	883.21 ± 253.53	1111.74 ± 340.13	< 0.001^**^
First 12-hour drainage (mL)	310.21 ± 313.29	473.55 ± 357.07	0.001^**^
Second 12-hour drainage (mL)	266.68 ± 164.39	342.07 ± 193.76	0.004^**^
Total drainage (24 hours) (mL)	576.89 ± 412.11	815.62 ± 431.79	< 0.001^**^
Re-exploration from potential bleeding or tamponade	3 (3.16)	6 (6.32)	0.306
Readmission due to reasons related to surgery	8 (8.42)	5 (5.26)	0.389
Conversion to full sternotomy	3 (3.16)	N/A	N/A
Reintubation	10 (10.53)	9 (9.47)	0.809
Mechanical ventilation > 24 hours	16 (16.84)	21 (22.11)	0.360
Mediastinitis	1 (1.05)	9 (9.47)	0.009^**^
Pacemaker	12 (12.63)	26 (27.37)	0.011^*^
Postoperative AKI	22 (23.16)	28 (29.47)	0.323
Hemodialysis	3 (3.16)	5 (5.26)	0.470
Postoperative renal failure	7 (7.37)	9 (9.47)	0.601
Pleural effusion requested drainage	14 (14.74)	11 (11.58)	0.520
Postoperative LVEF (%)	59.31 ± 8.75	54.78 ± 11.18	0.002^**^
Postoperative arrhythmia	35 (36.84)	33 (34.74)	0.762
In-hospital/30-day mortality	3 (3.16)	1 (1.05)	0.312

AKI=acute kidney injury; AVR=aortic valve replacement; ICU=intensive care unit; LVEF=left ventricular ejection fraction; MFS=median full sternotomy; MIAVR=minimally invasive aortic valve replacement; RAT=right anterior thoracotomy; RBC=red blood cells; SD=standard deviation

* *P*<0.05,

** *P*<0.01

### Operative Technique

**Fig. 2:**
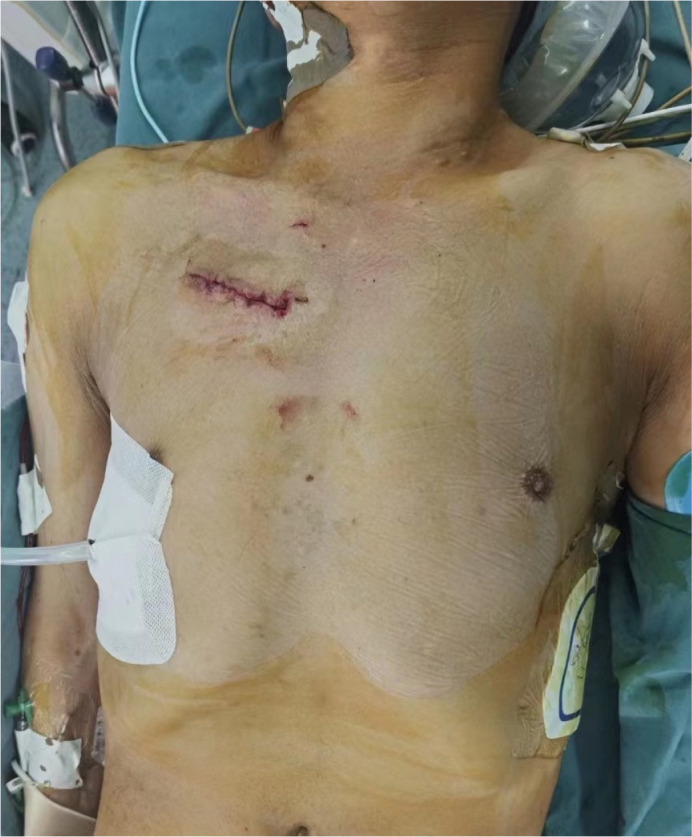
Surgical opening length.

**Fig. 3:**
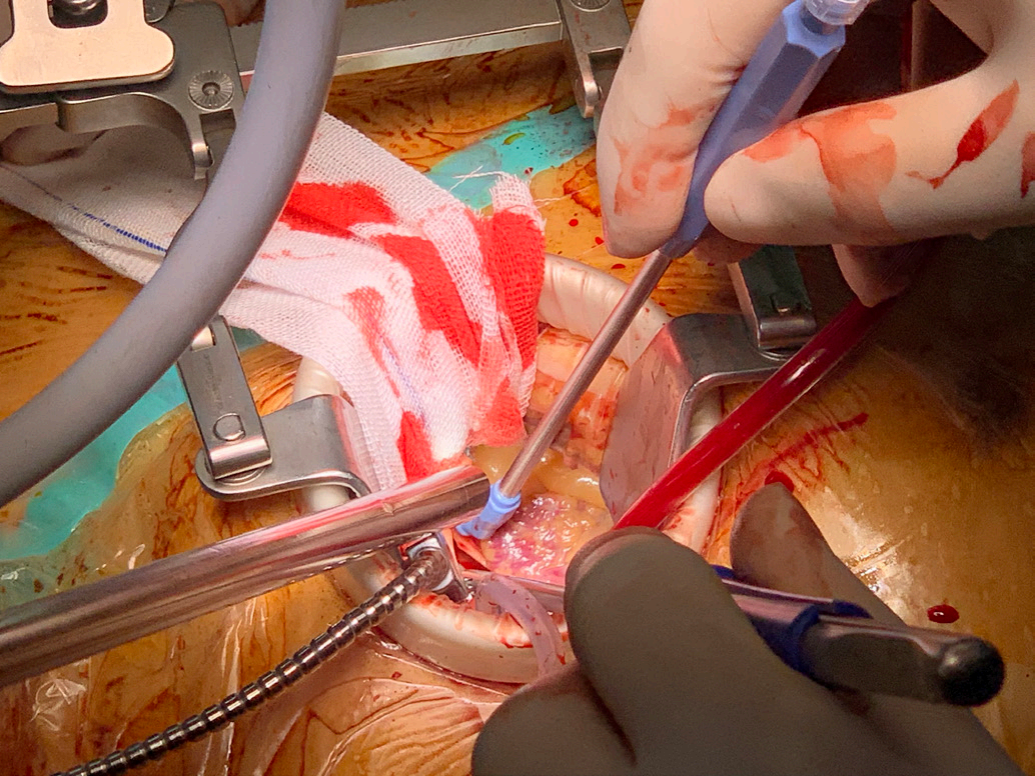
Minimally invasive aortic valve replacement surgical setup.

As described by Rao P.N. et al.^[10]^, the patient was positioned supine with a 3-7 cm elevation of the upper right-back chest region using a small pillow. A 5-7 cm incision was created in the femoral triangle region to facilitate catheterization of the femoral artery and vein. Cardiopulmonary bypass (CPB) was established through the femoral artery, femoral vein, and right internal jugular vein. A 5 cm right anterior intercostal skin incision served as the operative access point (Figure 2)^[1,2]^. Following body cooling to 30°C via CPB, the aorta was clamped at the aortic arch. Anterograde histidinetryptophan-ketoglutarate (HTK) cardioplegia was directly infused into the left and right coronary arteries, inducing cardiac arrest and providing myocardial protection (Figure 3). The pathological valve was excised and replaced. To eliminate air bubbles and avert reperfusion injury, a 50 ml needle was introduced into the ascending aorta between a pre-established sealing. Upon releasing the aortic clamp, an automated external defibrillator was utilized if spontaneous cardiac activity was not restored.

## RESULTS

### Preoperative Results

Before the implementation of PSM, the variables weight, BMI, recent dialysis, history of CAD, presence of functional mitral valve regurgitation (MVR), preoperative neurological disorders, and endocarditis displayed statistical significance. However, after PSM application, these variables were successfully balanced between the treatment groups. Additionally, the age variable exhibited a very low *P*-value, nearing the threshold of statistical significance, which was balanced as well in the matched group (Table 1). In the unmatched group, patients who underwent MFS-AVR surgery exhibited a higher mean age (50.49 ± 12.37 years old) compared to those who underwent RAT-MIAVR (47.53 ± 14.23 years old), with a *P*-value near the threshold (*P*=0.053). In the matched sample, no significant age difference was observed between the RAT-MIAVR and MFS-AVR groups (RAT-MIAVR: 47.37 ± 14.01 years old *vs.* MFS-AVR: 48.09 ± 13.69 years old, *P*=0.718). Prior to PSM, weight, identified as a potential confounding factor, demonstrated significant differences between the RAT-MIAVR and MFS-AVR groups (RAT-MIAVR: 67.33 ± 11.81 kg *vs.* MFS-AVR: 64.45 ± 10.95 kg, *P*=0.019); however, no significant differences were observed after PSM (RAT-MIAVR: 66.66 ± 11.46 kg *vs.* MFS-AVR: 67.16 ± 12.32 kg, *P*=0.775).

BMI was also considered a confounder, with a *P*-value approaching the significance level in both the unmatched group (RAT-MIAVR: 24.00 ± 2.94 kg/m^2^ *vs.* MFS-AVR: 23.05 ± 3.08 kg/m^2^, *P*=0.005) and the matched group (RAT-MIAVR: 23.79 ± 2.96 kg/m^2^ *vs.* MFS-AVR: 23.60 ± 3.27 kg/m^2^, *P*=0.677). In the unmatched group, preoperative recent dialysis was observed in three cases (2.80%) for RAT-MIAVR and one case (1.05%) for MFS-AVR, with a significant *P*-value at 0.012. In the matched groups, one case (1.05%) was reported in the RAT-MIAVR group, and no case was reported in the MFS-AVR group (*P*=0.316).

Before PSM, history of CAD was significant in the RAT-MIAVR group (RAT-MIAVR: 10 cases [9.35%] *vs.* MFS-AVR: 70 cases [18.97%], *P*=0.019); however, it was not significant after PSM (both groups: seven cases [7.37%], *P*=1.000). The presence of functional MVR was significant in the pre-PSM group (RAT-MIAVR: 10 cases [9.35%] *vs.* MFS-AVR: 69 cases [18.70%], *P*=0.022), but not in the post-PSM group (RAT-MIAVR: 10 cases [10.53%] *vs.* MFS-AVR: eight cases [8.42%], *P*=0.620). Preoperative neurological disorders, considered confounding factors, had a higher incidence in the unmatched RAT-MIAVR group (eight cases [7.48%]) compared to the MFS-AVR group (eight cases [2.17%]) (*P*=0.007). In the matched sample, the incidence was RAT-MIAVR: four cases (4.21%) *vs.* MFS-AVR: five cases (5.26%) (*P*=0.733).

Finally, PSM effectively eliminated the confounding effect of preoperative endocarditis. Prior to PSM, the unmatched MFS-AVR group had a significantly higher number of patients diagnosed with endocarditis (54 cases [14.63%]) compared to the RAT-MIAVR group (four cases [3.74%]) (*P*=0.002). No significant difference was observed between the two groups in the matched sample (RAT-MIAVR: four cases [4.21%] *vs.* MFS-AVR: two cases [2.11%], *P*=0.407). Other preoperative characteristics were not found to be significant.

### Intraoperative Results

The intraoperative results are presented in Table 2, encompassing CPB time, aortic cross-clamping (ACC) time, and overall operative time. The RAT-MIAVR group exhibited significantly longer CPB time (129.44 ± 30.63 minutes) compared to the MFS-AVR group (117.48 ± 36.11 minutes) (*P*=0.015), as well as a notably extended ACC time (76.41 ± 18.00 minutes) relative to the MFS-AVR group (68.18 ± 19.46 minutes) (*P*=0.003). Furthermore, the RAT-MIAVR group demonstrated a significantly protracted total operative time (356.65 ± 69.69 minutes) in contrast to the MFS-AVR group (322.05 ± 63.47 minutes) (*P*<0.001). However, regarding the valve size and valve type, our analysis revealed no statistically significant differences between the two groups.

**Table 2:** Matched intraoperative characteristics.

Variables	RAT-MIAVR (N=95)	MFS-AVR (N=95)	*P*-value
	Mean (± SD)/N (%)		
CPB time (min)	129.44 ± 30.63	117.48 ± 36.11	0.015^*^
ACC time (min)	76.41 ± 18.00	68.18 ± 19.46	0.003^**^
Operation time (min)	356.65 ± 69.69	322.05 ± 63.47	< 0.001^**^
Valve diameter (mm)	22.53 ± 2.06	22.67 ± 1.70	0.591
AV size on ultrasound (mm)	25.54 ± 3.81	26.15 ± 4.81	0.333
Mechanical valve	85 (89.47)	79 (83.16)	0.205
Biosynthetic valve	10 (10.53)	16 (16.84)	

ACC=aortic cross-clamping; AV=aortic valve; AVR=aortic valve replacement; CPB=cardiopulmonary bypass; MFS=median full sternotomy; MIAVR=minimally invasive aortic valve replacement; RAT=right anterior thoracotomy; SD=standard deviation

* *P*<0.05,

** *P*<0.01

### Postoperative Results

Table 3 presents the postoperative results for the matched groups. A significant decrease in the in-hospital stay was observed for the RAT-MIAVR group (14.78 ± 8.10 days) compared to the MFS-AVR group (17.97 ± 7.49 days) (*P*=0.005). Furthermore, the RAT-MIAVR group exhibited a significant reduction in blood loss after surgery (883.21 ± 253.53 mL) compared to the MFS-AVR group (1111.74 ± 340.13 mL) (*P*<0.001).

The MFS-AVR group demonstrated a significantly higher first 12-hour drainage volume after operation (473.55 ± 357.07 mL) compared to the RAT-MIAVR group (310.21 ± 313.29 mL) (*P*<0.001), as well as a higher second 12-hour drainage volume after operation (342.07 ± 193.76 mL *vs.* 266.68 ± 164.39 mL, *P*=0.004). The total drainage volume was also significantly higher in the MFS-AVR group (815.62 ± 431.79 mL) compared to the RAT-MIAVR group (576.89 ± 412.11 mL) (*P*<0.001).

The incidence of mediastinitis was significantly higher in the MFS-AVR group (nine cases, [9.47%]) compared to the RAT-MIAVR group (one case [1.05%]) (*P*=0.009). Additionally, the need for a pacemaker after surgery was significantly higher in the MFS-AVR group (25 cases [32.05%]) than in the RAT-MIAVR group (five cases [12.82%]) (*P*=0.025). Postoperative left ventricular ejection fraction (LVEF) was significantly higher in the RAT-MIAVR group (59.31 ± 8.75%) compared to the MFS-AVR group (54.78 ± 11.18%) (*P*=0.002). No statistically significant differences were observed in other postoperative findings.

## DISCUSSION

The main objective of our study was to compare the feasibility, safety, and efficacy of RAT-MIAVR with those of MFS-AVR. Our findings indicate that there were no significant differences in mortality and morbidity between the two approaches. Furthermore, we found that RAT-MIAVR was as feasible and safe as MFS-AVR. However, there were some limitations and unexplained findings associated with RAT-MIAVR that should be further explored.

RAT-MIAVR often requires a longer period for the body to regain its normal temperature after ACC, due to the use of peripheral cannulation to facilitate CPB. Prolonged CPB time during AVR could lead to challenging complications^[11,12]^. Although prolonged CPB, ACC, and total operative time were significant in our RATMIAVR group, we still did not observe any significant prolongation in mechanical ventilation, intensive care unit time, and total hospitalization time between the two groups. Similar studies also support that MIAVR with increased CPB time does not result in severe prolonged CPB time complications^[13-17]^. To explain this finding, we went through several recent pieces of research. Michael Robich et al.^[18]^ suggested that the prolonged use of CPB leads to increased serum soluble syndecan-1, indicating endothelial shedding. This shedding is linked to neutrophil mobilization out of the bone marrow leading to leukocytosis, which amplifies inflammation and tissue damage. However, a study conducted by Nicole A.M. et al.^[19]^ indicates that using heparin biocompatible coating during the CPB - a process implemented in our MIAVR protocol - may prevent the increase of syndecan-1 in serum blood. This mitigation could reduce the incidence of leukocytosis and systematic inflammation during CPB. Another possibility that may prevent CPB complications during RAT-MIAVR is the use of HTK cardioplegia. Plestis K. et al.^[20]^, in their study, pointed out that the use of HTK and Cor-Knot® titanium fastener could significantly improve postoperative complications and decrease intraoperative time{Plestis, 2018 #39@@hidden}{Plestis, 2018 #39} {Plestis, 2018 #39}. Interestingly, Mauro Del Giglio et al.^[14]^, in their research, reported that there was no significant increase in CPB time in their RAT-MIAVR module. Their finding may be attributable to the application of three running sutures during prosthetic valve fixation or potentially the use of sutureless (SU) valves in their RAT module. Those factors make replacing the valve easier and faster than conventional mechanical valves. Their results also showed no significant development in postoperative complications suggested by prolonged CPB. Yet, RAT+SU-AVR is still a relatively new technique, long-term life quality and valve life expectancy are still to be determined.

Postoperative in-hospital stay duration is an essential factor to consider, as shorter stays are often associated with reduced healthcare costs, lower risk of hospital-acquired infections, and improved patient satisfaction. In our study, RAT-MIAVR technique had a significantly shorter in-hospital stay after surgery compared to the MFS-AVR group. This suggests that patients who underwent RAT-MIAVR had experienced a faster recovery and were discharged earlier than those who underwent MFS-AVR. Similar findings have been reported in recent studies which demonstrated that MIAVR was associated with a shorter in-hospital stay compared to the conventional MFS-AVR approach^[17]^.

Nevertheless, the estimated blood loss and postoperative drainage were significant findings in this study. In our RAT-MIAVR approach, the significant decrease in blood loss provides remarkable evidence of a significant reduction in cellular injury and improved recovery. However, existing literature further corroborates that MIAVR procedures necessitate fewer blood transfusions in comparison to traditional methods^[21]^.

Mediastinitis is a serious complication of MFS-AVR, with reported mortality rates ranging from 12% to 47%^[22]^. While other studies have demonstrated absence of mediastinitis in MIAVR^[21]^, our research revealed a low incidence of mediastinitis in RAT-MIAVR, with only one case detected in 2016. This event was attributed to inadequate pericardial drainage, leading to the retention of fluid and subsequent pericardial effusion. We have since modified our technique by enlarging the pericardial opening to ensure adequate drainage and avoid this complication in subsequent surgeries. Our findings suggest that RAT-MIAVR may have a lower risk of mediastinitis compared to MFS-AVR, and proper drainage techniques are essential to prevent this serious complication. The present investigation revealed that a high percentage of patients undergoing MFS-AVR required pacemaker utilization (32%), which may imply the presence of severe arrhythmia or heart block. However, our study did not detect significant variations in the overall incidence of arrhythmia between the MFS-AVR and RATMIAVR groups. This result may be attributed to the prophylactic installation of pacemakers in patients who were deemed to be at high risk of developing arrhythmias postoperatively. However, it is important to note that in some cases, the pacemakers were not ultimately utilized.

LVEF is an essential measure of the heart’s pumping capacity, specifically assessing the percentage of blood expelled from the left ventricle during each contraction. An improvement in LVEF following surgery may indicate enhanced cardiac function. The study results demonstrated a significantly higher postoperative LVEF in the RAT-MIAVR group compared to the MFS-AVR group. This finding suggests that patients who underwent RAT-MIAVR experienced superior postoperative cardiac function relative to those who underwent MFS-AVR. Recent studies have reported similar findings. Glauber et al. discovered that MIAVR was associated with improved postoperative LVEF compared to conventional sternotomy procedures^[23]^.

Although the RAT-MIAVR approach offers benefits such as smaller incisions and improved cosmetic outcomes, its use is limited by the prevalence of vascular disorders in older patients with aortic valve disease. Specifically, atherosclerotic plaques, thrombosis, and inflammatory vesicular disease in the femoral vessels can pose risks during retrograde CPB perfusion used in RAT-MIAVR surgery^[24]^. To mitigate these risks, our center employs multidetector computed tomography scans to evaluate the entire aorta, femoral arteries, and internal carotid artery for enabling the identification of conditions such as ulcers, aortic dissections, aneurysms, and severe calcifications. Patients found to have decreased vascular diameter or intervascular disease are recommended for MFS-AVR surgery to avoid the risk of complications associated with peripheral vascular disease.

Overall, our matched groups had relatively mild disease severity. Our preoperative results showed that the mean age of patients in both groups did not exceed 61 years. Older patients were eligible for elective transcatheter aortic valve replacement surgery. These results support the growing body of evidence suggesting that MIAVR techniques, such as RAT-MIAVR, may be associated with better postoperative cardiac function compared to conventional MFS-AVR. Further research and larger randomized controlled trials are needed to confirm these findings and to determine the longterm implications of these differences in LVEF.

### Limitations

This study has limitations due to its retrospective nature and single-institution setting, which may limit the generalizability of the results to other populations. Additionally, the limited follow-up period prevented a comprehensive assessment of the long-term outcomes of MIAVR approach.

## CONCLUSION

In conclusion, RAT-MIAVR is a relatively new surgical technique that requires further investigation to ascertain its safety and feasibility in AVR treatment. Our study demonstrates that RAT-MIAVR is a feasible, safe, and effective alternative to MFS-AVR. Despite the limitations associated with RAT-MIAVR, our results indicate no significant difference in mortality and morbidity between the two approaches. The RAT-MIAVR group exhibited a shorter in-hospital stay, reduced blood loss, improved postoperative cardiac function, as evidenced by higher LVEF, and superior cosmetic results due to smaller incisions and less scarring. Moreover, RAT-MIAVR appears to have a lower risk of mediastinitis compared to MFS-AVR when proper drainage techniques are employed.

The use of RAT-MIAVR in patients with vascular disorders remains limited, and more research is needed to address this challenge. Our findings contribute to the growing evidence supporting the advantages of MIAVR techniques, such as RAT-MIAVR. However, larger randomized controlled trials or meta-analyses are required to confirm these findings and to determine the long-term implications of the observed differences in postoperative cardiac function and cosmetic outcomes.

**Table:** 

Author's Roles & Responsibilities	
AOKA	Substantial contributions to the conception or design of the work; or the acquisition, analysis, or interpretation of data for the work; drafting the work or revising it critically for important intellectual content; final approval of the version to be published
RL	Drafting the work or revising it critically for important intellectual content; agreement to be accountable for all aspects of the work in ensuring that questions related to the accuracy or integrity of any part of the work are appropriately investigated and resolved; final approval of the version to be published
CHL	Substantial contributions to the conception or design of the work; or the acquisition, analysis, or interpretation of data for the work; final approval of the version to be published
AMZ	Substantial contributions to the conception or design of the work; or the acquisition, analysis, or interpretation of data for the work; final approval of the version to be published
XW	Agreement to be accountable for all aspects of the work in ensuring that questions related to the accuracy or integrity of any part of the work are appropriately investigated and resolved; final approval of the version to be published

## References

[1] Vahanian A, Alfieri O, Andreotti F, Antunes MJ, Joint Task Force on the Management of Valvular Heart Disease of the
European Society of Cardiology (ESC), European Association for Cardio-Thoracic Surgery (EACTS) (2012). Guidelines on the management of valvular heart disease (version
2012). Eur Heart J.

[2] Campo J, Tsoris A, Kruse J, Karim A, Andrei AC, Liu M (2019). Prognosis of severe asymptomatic aortic stenosis with and without
surgery. Ann Thorac Surg.

[3] Brown JM, O'Brien SM, Wu C, Sikora JA, Griffith BP, Gammie JS. (2009). Isolated aortic valve replacement in North America comprising
108,687 patients in 10 years: changes in risks, valve types, and outcomes in
the society of thoracic surgeons national database. J Thorac Cardiovasc Surg.

[4] Cosgrove DM 3rd, Sabik JF. (1996). Minimally invasive approach for aortic valve
operations. Ann Thorac Surg.

[5] Rodríguez JE, López MJ, Carrascal Y, Maroto LC, Forteza A, Cortina J (1996). Sustitución valvular aórtica por
miniesternotomía. Rev Esp Cardiol.

[6] Schwartz DS, Ribakove GH, Grossi EA, Stevens JH, Siegel LC, St Goar FG (1996). Minimally invasive cardiopulmonary bypass with cardioplegic
arrest: a closed chest technique with equivalent myocardial
protection. J Thorac Cardiovasc Surg.

[7] Detter C, Deuse T, Boehm DH, Reichenspurner H, Reichart B. (2002). Midterm results and quality of life after minimally invasive vs.
conventional aortic valve replacement. Thorac Cardiovasc Surg.

[8] Mächler HE, Bergmann P, Anelli-Monti M, Dacar D, Rehak P, Knez I (1999). Minimally invasive versus conventional aortic valve operations: a
prospective study in 120 patients. Ann Thorac Surg.

[9] Austin PC. (2011). Optimal caliper widths for propensity-score matching when
estimating differences in means and differences in proportions in
observational studies. Pharm Stat.

[10] Rao PN, Kumar AS. (1993). Aortic valve replacement through right
thoracotomy. Tex Heart Inst J.

[11] Martins RS, Ukrani RD, Memon MK, Ahmad W, Akhtar S. (2021). Risk factors and outcomes of prolonged cardiopulmonary bypass
time in surgery for adult congenital heart disease: a single-center study
from a low-middle-income country. J Cardiovasc Surg (Torino).

[12] Madhavan S, Chan SP, Tan WC, Eng J, Li B, Luo HD (2018). Cardiopulmonary bypass time: every minute counts. J Cardiovasc Surg (Torino).

[13] Oo S, Khan A, Chan J, Juneja S, Caputo M, Angelini G (2023). Propensity matched analysis of minimally invasive versus
conventional isolated aortic valve replacement. Perfusion.

[14] Del Giglio M, Mikus E, Nerla R, Micari A, Calvi S, Tripodi A (2018). Right anterior mini-thoracotomy vs. conventional sternotomy for
aortic valve replacement: a propensity-matched comparison. J Thorac Dis.

[15] Hancock HC, Maier RH, Kasim A, Mason J, Murphy G, Goodwin A (2021). Ministernotomy versus conventional sternotomy for aortic valve
replacement: a randomised controlled trial. BMJ Open.

[16] Shehada SE, Elhmidi Y, Mourad F, Wendt D, El Gabry M, Benedik J (2017). Minimal access versus conventional aortic valve replacement: a
metaanalysis of propensity-matched studies. Interact Cardiovasc Thorac Surg.

[17] Bruno P, Cammertoni F, Rosenhek R, Mazza A, Pavone N, Iafrancesco M (2019). Improved patient recovery with minimally invasive aortic valve
surgery: a propensity-matched study. Innovations (Phila).

[18] Robich M, Ryzhov S, Kacer D, Palmeri M, Peterson SM, Quinn RD (2020). Prolonged cardiopulmonary bypass is associated with endothelial
glycocalyx degradation. J Surg Res.

[19] Dekker NAM, Veerhoek D, van Leeuwen ALI, Vonk ABA, van den Brom CE, Boer C. (2020). Microvascular alterations during cardiac surgery using a heparin
or phosphorylcholine-coated circuit. J Cardiothorac Vasc Anesth.

[20] Plestis K, Orlov O, Shah VN, Wong J, Thomas M, Aharon A (2018). Facilitating technologies in minimally invasive aortic valve
replacement: a propensity score analysis. Interact Cardiovasc Thorac Surg.

[21] Paparella D, Santarpino G, Moscarelli M, Guida P, De Santis A, Fattouch K (2021). Minimally invasive aortic valve replacement: short-term efficacy
of sutureless compared with stented bioprostheses. Interact Cardiovasc Thorac Surg.

[22] Ang LB, Veloria EN, Evanina EY, Smaldone A. (2012). Mediastinitis and blood transfusion in cardiac surgery: a
systematic review. Heart Lung.

[23] Glauber M, Ferrarini M, Miceli A. (2015). Minimally invasive aortic valve surgery: state of the art and
future directions. Ann Cardiothorac Surg.

[24] Attia RQ, Hickey GL, Grant SW, Bridgewater B, Roxburgh JC, Kumar P (2016). Minimally invasive versus conventional aortic valve replacement:
a propensity-matched study from the UK national data. Innovations (Phila).

